# Cloacal reconstruction after a complex treatment of perineal haemangioma in a variant of PELVIS syndrome

**DOI:** 10.1186/s12887-015-0469-6

**Published:** 2015-10-08

**Authors:** Algirdas Zalimas, Gintas Posiunas, Sigitas Strupas, Ramunas Raugalas, Juozas Raistenskis, Gilvydas Verkauskas

**Affiliations:** Faculty of Medicine, Vilnius University, M.K. Ciurlionio Street 21, 03101 Vilnius, Lithuania; Children’s Surgery Centre, Faculty of Medicine, Vilnius University, Santariskiu Street 7, 08406 Vilnius, Lithuania; Department of Neurology and Neurosurgery, Vilnius University, Santariskiu Street 7, 08406 Vilnius, Lithuania; Department of Rehabilitation, Physical and Sports Medicine, Vilnius University, Santariskiu Street 7, 08406 Vilnius, Lithuania

**Keywords:** Perineal hemangioma, PELVIS syndrome, Cloaca, Laser treatment, Reconstruction

## Abstract

**Background:**

PELVIS is an acronym defining the association of perineal hemangioma, malformations of external genitalia, lipomyelomeningocele, vesicorenal abnormalities, imperforate anus and skin tag. Eleven cases have been reported according to the Orphanet data. Acronyms of LUMBAR and SACRAL syndrome have been used and most probably represent a spectrum of the same entity. Very little is known about the success and timing of cloacal reconstruction after the treatment of hemangioma. We present a variant of PELVIS syndrome and discuss the possibilities and optimal timing of surgical reconstruction.

**Case presentation:**

Female infant was born with persistent cloaca and multiple hemangiomas of genitals, perineal area and left thigh. Colostomy was performed after birth. In order to treat hemangioma and to make the reconstruction of cloaca possible, corticosteroid treatment orally and multiple laser treatments were performed alternating Nd:YAG laser and pulsed dye laser therapy. Cystoscopy confirmed hemangiomatosis in the mucosa of the common channel, bladder neck and septate vagina. Oral propranolol treatment was started at the age of 18 months and continued for 1 year. It induced rapid improvement of hemangiomas. Two more pulsed dye laser treatments were performed to remove residuals of hemangiomas from the perineum and genital area. Posterior sagital reconstruction by separation of the rectum, mobilization of urogenital sinus and vaginal reconstruction was performed with no major bleeding at the age of 4 years. Postoperatively, after a period of progressive rectal dilatation colostomy was closed. Girl is now 6 years old, dry day and night without residual urine and normal upper tracts. Rectal calibration is normal, fecal continence is still to be evaluated but constipation is easily manageable. CT of the spine and the perineum showed sacral dysplasia and spina bifida with lumbo-sacral lipoma and tethering of terminal filum without neurological deterioration at the moment but requiring close neurological monitoring.

**Conclusions:**

Large perineal hemangiomas are commonly associated with extracutaneous abnormalities. Successful reconstructive surgery is possible after significant reduction of hemangioma by complex treatment.

## Background

Infantile hemangiomas are sometimes complicated and associated with systemic malformations. PELVIS is an acronym defining the association of perineal hemangioma, malformations of external genitalia, lipomyelomeningocele, vesicorenal abnormalities, imperforate anus and skin tag [1]. Eleven cases have been reported according to the Orphanet data. Acronyms of LUMBAR and SACRAL syndrome have been used and most probably represent a spectrum of the same entity. Very little is known about the success of surgical reconstruction of anomalies complicated by haemangioma in these cases.

We report the 2 years follow-up results of cloacal reconstruction in a girl with PELVIS syndrome. The manuscript was performed with the approval of the Bioethics Committee of the Childrens Hospital, affiliate Vilnius University Hospital Santariskiu clinics and is in compliance with the Helsinki Declaration.

## Case presentation

**Fig. 1:**
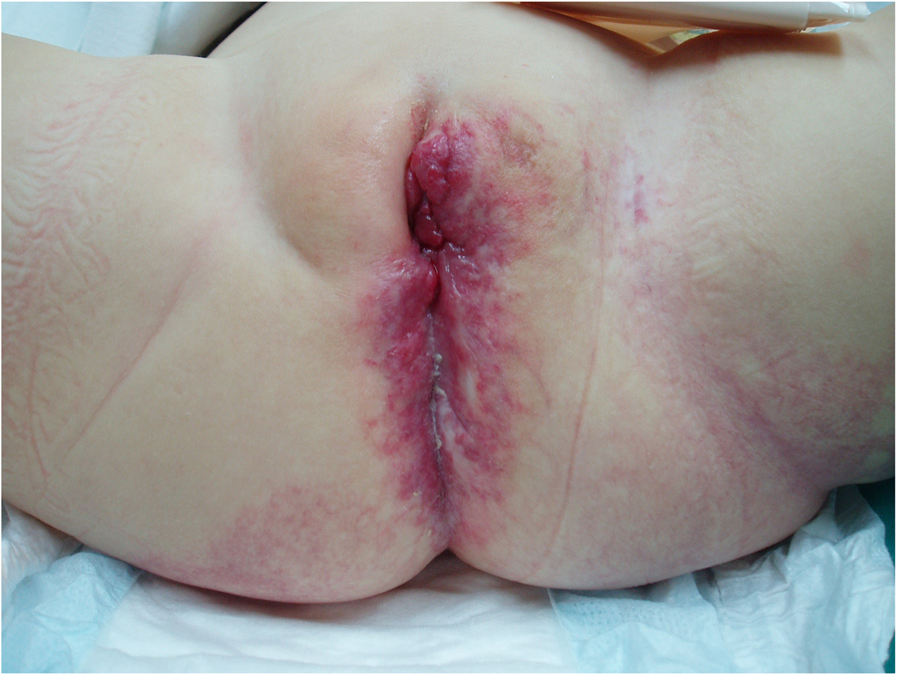
Residuals of hemangiomas of genitals, perineal area and left thigh at 3 years of age

Female infant was born with persistent cloaca and multiple hemangiomas of genitals, perineal area and left tight (Fig. 1). Colostomy was performed after birth. During the first month of life aggressive proliferation of hemangiomas was observed. Combined treatment was administered: corticosteroid treatment 15 mg per day orally and laser therapy by Nd:YAG laser 1064 nm ice cube method on hemangiomas of the perineal area and pulsed dye laser therapy 595 nm on hemangiomas of left thigh. After 2 months the dose of prednisolone was reduced to 5 mg, but proliferation of hemangiomas was observed and laser therapy was performed again by long pulse Nd:YAG laser on genitals and pulsed dye laser therapy on perineal area and left thigh. In order to make the reconstruction of cloaca possible, laser treatments were repeated by pulsed dye laser 4 more times. Good effect was observed externally but cystoscopy showed prominent hemangiomas in the mucosa of cloaca and the bladder neck. Oral propranolol treatment was administered 2 mg/kg per day at the age of 18 months, followed by rapid improvement of hemangiomas. Pulsed dye laser treatment was performed twice more during 4 month period removing residuals of hemangiomas from the perineum and genital area. Propranolol treatment was continued for 8 months until almost no hemangiomas were seen and reconstructive operation was considered to be safe. Posterior sagital reconstruction by separation of the rectum, mobilization of urogenital sinus and vaginal reconstruction was performed with no major bleeding at the age of 4 years. Postoperatively, after a period of progressive rectal dilatation colostomy was closed. Girl is now 6 years old. Urinary continence was evaluated by a diary and the ultrasound of kidneys and the bladder. She is urinary continent: dry day and night, voiding without residual urine in the bladder and no dilatation of the upper tract. Fecal continence is still to be evaluated but bowel movements are regular with short periods of constipation manageable with laxatives and/or enemas occasionally. CT of spine and the perineum showed sacral dysplasia and spina bifida with lumbo-sacral lipoma and tethering of terminal filum without neurological deterioration at the moment but requiring close neurological monitoring.

## Discussion

Due to the extreme rarity there is no treatment algorithm of this syndrome. Every individual case dictates timing and the choice of therapy for the best long term outcome. Congenital anomalies of a urogenital tract, like persistent cloaca, are by itself associated with a great chance of poor functional outcome even after successful surgical procedure. The association of a disfiguring haemangioma in the area of reconstruction gives first impression of poorer prognosis.

The recent discovery of propranolol effectiveness and the improved employment of Laser technologies may improve the perspective for these children.

Léauté-Labrèze *et al*. reported the antiproliferative effect of propranolol on infantile hemangiomas on June 2008 [2]. Recent meta-analysis demonstrated the corticosteroid studies to have a pooled response rate of 69 % versus the propranolol response rate of 97 % (*p* < 0.001) [3]. Propranolol introduction in the treatment complex of our case also seemed to induce marked improvement.

Laser therapy for proliferating hemangiomas is controversial. One study have demonstrated good results with either the 585 nm or 595 mm pulsed-dye laser on superficial but not deep infantile hemangiomas [4]. Other studies showed no difference between complete and nearly complete clearance with early laser treatment compared to observation alone at 1 year of age [5]. Severe ulceration and scarring have been reported as an adverse event, particularly when treating segmental hemangiomas during the proliferative phase.

With our choice of Nd:YAG laser 1064 nm ice cube method and pulsed dye laser therapy 595 nm we didn’t observe complications and the growth of hemangiomas has stopped and regression induced. Combined treatment-systemic therapy and multiple laser applications seems to be the most effective treatment.

Reconstructive operation mobilizing rectum and urogenital sinus and performing genitoplasty by posterior sagital approach in this case requires extensive dissection and mobilization of delicate structures what could be harassed by bleeding [6]. That is why surgery which is advocated at the age from 6 months to 1 year was postponed to 4 years of age. It allowed carrying out the difficult surgery successfully with minimal blood loss and no long term complications.

## Conclusion

we support the current evidence of common association between large perineal hemangiomas and extracutaneous anomalies. Delaying reconstructive surgery until the safe reduction of hemangiomas by complex treatment does not seem to worsen long term functional and cosmetic results.

## Consent

Written informed consent was obtained from the parent of the patient for publication of this Case report and any accompanying images. A copy of the written consent is available for review by the Editor of this journal.

## References

[1] Girard C, Bigorre M, Guillot B, Bessis B (2006). PELVIS syndrome. Arch Dermatol.

[2] Léauté-Labrèze C, Dumas De la Roque E, Hubiche T (2008). Propranolol for severe hemangiomas of infancy. N Engl J Med.

[3] Izadpanah A, Izadpanah A, Kanevsky J, Belzile E, Schwarz K (2013). Propranolol versus Corticosteroids in the treatment of infantile hemangioma: a systematic review and meta-analysis. Plast Reconstr Surg.

[4] Kolde G (2003). Early pulsed-dye laser treatment of childhood haemangiomas. Lancet.

[5] Michel JL (2003). Treatment of hemangiomas with 595 nm pulsed dye laser dermobeam. Eur J Dermatol.

[6] Levitt M, Peña A (2010). Cloacal malformations: lessons learned from 490 cases. Seminars in Pediatric Surgery.

